# Brain Imaging of the GLP-1 Receptor in Obesity Using ^68^Ga-NODAGA-Exendin-4 PET

**DOI:** 10.3390/brainsci11121647

**Published:** 2021-12-15

**Authors:** Laura N. Deden, Jan Booij, Joanes Grandjean, Judith R. Homberg, Eric J. Hazebroek, Martin Gotthardt, Marti Boss

**Affiliations:** 1 Department of Medical Imaging, Radboud University Medical Center, 6525 GA Nijmegen, The Netherlands; ldeden@rijnstate.nl (L.N.D.); jan.booij@radboudumc.nl (J.B.); Joanes.Grandjean@radboudumc.nl (J.G.); marti.boss@radboudumc.nl (M.B.); 2 Department of Surgery, Vitalys Clinic, Rijnstate Hospital, 6815 AD Arnhem, The Netherlands; ehazebroek@rijnstate.nl; 3 Department of Radiology and Nuclear Medicine, Amsterdam University Medical Centers, 1105 AZ Amsterdam, The Netherlands; 4 Center for Medical Neuroscience, Department of Cognitive Neuroscience, Donders Institute for Brain, Cognition and Behaviour, Radboud University Medical Center, 6525 EN Nijmegen, The Netherlands; judith.homberg@radboudumc.nl; 5 Division of Human Nutrition and Health, Wageningen University, 6708 PB Wageningen, The Netherlands

**Keywords:** GLP-1 receptor, PET, obesity, brain, ^68^Ga-NODAGA-exendin-4

## Abstract

Stimulation of glucagon-like peptide-1 (GLP-1) receptors increases the insulin release in the pancreas during high glucose levels, and also stimulates a feeling of satiety. Likewise, synthetic GLP-1 receptor agonists derived from exendin are used successfully in the treatment of type-2 diabetes mellitus and obesity. Interestingly, preclinical and clinical studies further suggest that GLP-1 receptor agonists may decrease motor, behavioral, and cognitive symptoms in (animal models) Parkinson’s disease and Alzheimer’s disease and may slow down neurodegeneration. These observations suggest stimulation of GLP-1 receptors in the brain. The GLP-1 positron emission tomography (PET) tracer ^68^Ga-NODAGA-exendin-4 has been developed and successfully used for imaging in humans. In an ongoing study on the effects of bariatric surgery on GLP-1 receptor expression, we performed ^68^Ga-NODAGA-exendin-4 PET in obese subjects. Here we evaluated whether GLP-1 receptor binding could be visualized in the central nervous system in 10 obese subjects (seven woman; body mass index: mean ± SD: 39 ± 4.4 kg/m^2^) before bariatric surgery. Although we observed clear uptake in the pituitary area (mean SUV_max_ 4.3 ± 2.3), we found no significant uptake in other parts of the brain. We conclude that ^68^Ga-NODAGA-exendin-4 PET cannot be used to analyze GLP-1 receptors in the brain of obese subjects.

## 1. Introduction

Glucagon-like peptide-1 (GLP-1) is predominantly synthesized in enteroendocrine cells of the distal small intestine and secreted in the blood when food enters the duodenum (for a recent review, see Mori et al. [1]). GLP-1 belongs to the incretin family (INtestinal seCRETion of INSulin). Incretins increase the release of insulin in the pancreas during high blood glucose levels, which is named the “incretin effect” [2].

GLP-1 is also synthesized in the brain, and particularly in the nucleus tractus solitarius, which is located in the brainstem [3]. These GLP-1 expressing neurons extend to parts of the hypothalamus, particularly the paraventricular nucleus and arcuate nucleus [4]. GLP-1 secretion stimulates a feeling of saturation, and as such plays a role in the regulation of appetite. Likewise, synthetic GLP-1 receptor agonists are used successfully in the treatment of type-2 diabetes mellitus and to reduce overweight in obesity (for recent reviews, see Nauck and Meier [5] and Hussein et al. [6]).

GLP-1 receptors are, however, not only located in the hypothalamus and nucleus tractus solitarius, but also in the substantia nigra, amygdala, hippocampus as well as in cortical areas such as the lateral prefrontal cortex [4,7,8,9,10,11]. Stimulation of these receptors may induce synaptogenesis/neurogenesis and protect against oxidative stress, neuroinflammation, and apoptosis [12]. Interestingly, in animal models of Parkinson’s disease (PD) and Alzheimer’s disease (AD), administration of GLP-1 receptor agonists may decrease the amyloid load, slow down dopaminergic degeneration, and improve motor and cognitive functions such as learning and memory [8,12,13,14,15,16,17,18,19,20,21,22,23].

The half-life of GLP-1 is short (a few minutes) because it is rapidly metabolized by the enzyme dipeptidyl peptidase IV (DDP-IV) [24]. Fortunately, the naturally occurring GLP-1 agonist exendin-4 is resistant to DDP-IV degradation. Exendin-4 has a much longer half-life (approximately 2.4 h) [25] than GLP-1 and is thus suitable for treating type-2 diabetes mellitus by increasing insulin secretion. Several synthetic GLP-1 agonists derived from exendin-4 have been produced successfully [26].

Importantly, a recent randomized, placebo-controlled study showed that administration of the GLP-1 agonist exenatide (a synthetic GLP-1 analog derived from exendin-4) is able to slow down motor progression in patients suffering from PD [27]. Moreover, a smaller, open label study showed significant effects on cognition in PD [28]. Additionally, a small randomized, placebo-controlled study on AD showed that exenatide prevented decline of cerebral glucose metabolism, measured with ^18^F-FDG positron emission tomography (PET) [29].

PET tracers derived from exendin have been developed successfully, including ^68^Ga-NODAGA-exendin-4 (for a recent review, see Jansen et al. [30]). In a recent study, we demonstrated the ability of ^68^Ga-NODAGA-exendin-4 to visualize GLP-1 receptor expression on pancreatic beta cells [31]. Since GLP-1 receptor agonists are successfully used to reduce body weight and improve insulin resistance in obese subjects, and bariatric surgery may influence GLP-1 levels [32], we recently started a clinical study on the effects of bariatric surgery on GLP-1 binding as measured with ^68^Ga-NODAGA-exendin-4 PET.

Although it is undisputed that GLP-1 agonists have central effects, the localization of GLP-1 receptors in the brain in human and mechanisms of GLP-1 agonists to cross the blood–brain barrier (BBB) are debated, especially since several observations do not support that these agonists can cross the BBB by general BBB permeability [4,9,33]. To contribute to this relevant issue, we here evaluated whether ^68^Ga-NODAGA-exendin-4 PET can be used to visualize and quantify GLP-1 receptors in the central nervous system (CNS). Furthermore, we discuss possible mechanisms of GLP-1 agonists to enter the CNS.

## 2. Materials and Methods

### 2.1. Participants

Study subjects have been participating in an ongoing clinical study on the effects of bariatric surgery on GLP-1 receptor expression at the Radboud University Medical Center (Radboud UMC, Nijmegen, The Netherlands). Inclusion criteria for participation in this study were obesity (body mass index (BMI) ≥ 35 kg/m^2^), age > 18 years, scheduled for bariatric surgery, and type-2 diabetes mellitus treated with insulin, sulfonylureas, or metformin. Exclusion criteria included pregnancy or breast feeding, kidney failure, liver failure, treatment with GLP-1 receptor agonists or DDP IV inhibitors, and BMI ≥ 50 kg/m^2^. Subjects underwent a medical evaluation, including medical history, physical examination, and blood tests, before study participation.

Participating subjects were eligible for the present analysis if they underwent whole-body ^68^Ga-NODAGA-exendin-4 PET imaging before surgery. All procedures were approved by the medical ethics committee of the Radboud UMC, and all subjects provided written informed consent in accordance with the Declaration of Helsinki.

### 2.2. Anthropometric Measurements

Demographic and anthropometric parameters were measured and documented by trained medical doctors.

### 2.3. PET Imaging

To measure GLP-1 receptor expression, subjects underwent PET imaging after an overnight fast or fasting for at least four hours. Imaging was performed 60 min after intravenous bolus injection of approximately 100 MBq ^68^Ga-NODAGA-exendin-4 PET (for details on the synthesis of the radiotracer, see Boss et al. [31]). PET imaging from the skull to pelvis was performed on a Biograph mCT-40 time-of-flight PET-CT scanner (Siemens) located at the Radboudumc.

After acquiring a low-dose CT scan, static images were acquired for 5 min per bed position. Low-dose CT (40 mAs, 130 kV) was obtained without contrast and applied for attenuation correction and anatomical localization. PET was reconstructed using ultrahigh definition, time-of-flight, scatter and attenuation correction and post-reconstruction Gaussian filter of 3 mm FWHM settings.

### 2.4. Volume-of-Interest (VOI) Analysis

Specific ^68^Ga-NODAGA-exendin-4 binding was assessed in the whole brain (excluding the pituitary), pituitary, and pancreas. Blood pool activity was determined in the left ventricle and uptake in liver and subcutaneous adipose tissue was used to assess background activity. Tracer uptake was determined in ellipsoid volumes of interest (VOI) that were representative for the whole organ and quantified using Inveon Research Workplace (version 4.1; Siemens Healthcare). Within the VOIs, the maximum standardized uptake (SUV_max_) and mean SUV (SUV_mean_) values were determined. Calculation of SUV was carried out as activity concentration (kBq/g)/[injected dose (MBq)/body weight (g)]; corrected for decay and assuming that 1 g tissue is equal to a volume of 1 mL.

### 2.5. Statistical Analysis

Since we analyzed data in 10 subjects with obesity, only descriptive statistics were performed.

## 3. Results

### 3.1. Study Subjects

We included 10 subjects (seven woman) with a median age of 55 years and a mean BMI of 39 kg/m^2^ (Table 1).

### 3.2. PET Analysis

**Figure 1:**
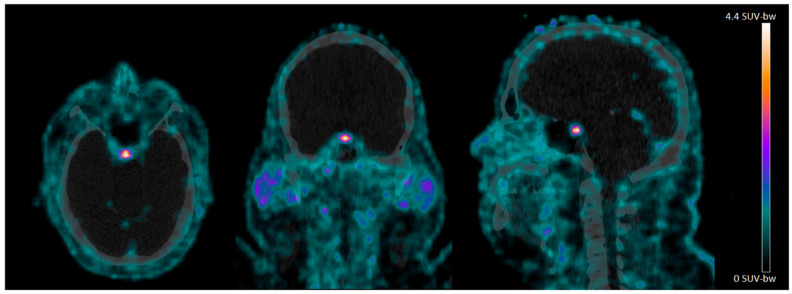
^68^Ga-NODAGA-exendin-4 PET/CT scans of the head acquired 60 min after injection in an obese subject. Note the intense uptake in the pituitary.

In the CNS, we observed clear ^68^Ga-NODAGA-exendin-4 uptake in the pituitary area (Figure 1), which was variable between subjects, with a mean SUV_mean_ of 1.7 ± 0.57 (range: 0.88–2.7) and mean SUV_max_ of 4.3 ± 3.3 (range: 1.4–9.1). However, no ^68^Ga-NODAGA-exendin-4 uptake was visible in other parts of the brain (mean SUV_mean_: 0.0010 ± 0.0066 (range: 0.0001–0.21) and SUV_max_ of 0.17 ± 0.095 (range: 0.013–0.32)) (Table 2).

**Figure 2:**
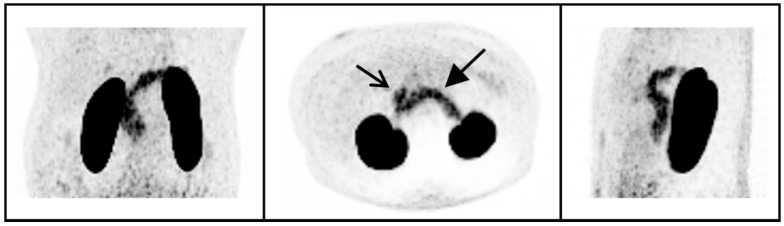
^68^Ga-NODAGA-exendin-4 PET maximum intensity projections of the abdomen of one subject in a coronal, axial, and sagittal view (left, middle, and right panel, respectively). Uptake in the pancreas (closed arrow), duodenum (open arrow) and kidneys can be observed.

In peripheral organs, clear uptake of ^68^Ga-NODAGA-exendin-4 was visible in the pancreas (Figure 2), with an average SUV_mean_ of 5.5 ± 1.8 (range 2.3–8.1) and SUV_max_ of 10.3 ± 3.0 (range: 5.0–15.5). In the blood pool, average SUV_mean_ was 1.5 ± 0.23 and in background tissues highest uptake levels were measured in the liver (SUV_mean_: 0.67 ± 0.17) and lowest in subcutaneous adipose tissue (SUV_mean_: 0.20 ± 0.054).

## 4. Discussion

In this study, we show that there is no significant uptake of ^68^Ga-NODAGA-exendin-4 in the brain (parts inside the blood–brain barrier; BBB) of subjects with obesity, although there is clear uptake in the pituitary. We also reproduce the accumulation of ^68^Ga-NODAGA-exendin-4 PET in the pancreas [31].

### 4.1. ^68^Ga-NODAGA-Exendin-4 Uptake in Peripheral Organs

We have confirmed the ability of ^68^Ga-NODAGA-exendin-4 to assess GLP-1 binding in the pancreas [31]. In line with this finding, other radiotracers derived from exendin showed the ability to visualize the GLP-1 receptor expression in the pancreas [30,34].

### 4.2. ^68^Ga-NODAGA-Exendin-4 Uptake in the Pituitary Area

We can show significant ^68^Ga-NODAGA-exendin-4 uptake in the pituitary of subjects with obesity. Since earlier studies showed the ability of ^68^Ga-NODAGA-exendin-4 PET to visualize GLP-1 receptors in beta-cells of the pancreas [31], and the pituitary is located outside the blood–brain barrier (BBB) and expresses GLP-1 receptors extensively in rats and humans, particularly in the neuro-pituitary [35,36], this was an expected finding. As GLP-1 receptors play an important role in appetite and stress regulation, and the pituitary is an essential part of the hypothalamus–pituitary–adrenal gland (HPA)-axis, the GLP-1 receptor expression in the pituitary may play a role in appetite and stress regulation in humans [37,38,39]. Importantly, GLP-1 receptor tracers such as ^68^Ga-NODAGA-exendin-4 offer the unique opportunity to test this postulate directly in humans.

The basal part of the hypothalamus is closely located to the pituitary. GLP-1 receptors are expressed in the paraventricular nucleus and arcuate nucleus [4,9], which are both located in the basal hypothalamus. The median eminence is a small brain area forming both the structural and functional bridge between the hypothalamus and the pituitary [40]. In the basal part of the hypothalamus and the median eminence, the blood–brain barrier (BBB) is not as tight as in other regions of the brain but rather “leaky”, due to fenestrated capillaries. The entry of hormones and other compounds into the brain, through fenestrated capillaries may be important for feedback regulation of, amongst others, the HPA-axis [41]. In other words, this special connection between the basal hypothalamus and the (neuro)pituitary may play a crucial role to integrate changes in hormones relevant for metabolism (like GLP-1 in blood) and food intake, with feedback systems within the brain (e.g., feeling of satiety).

In the present study, we were not able to differentiate binding to the pituitary from binding in the basal hypothalamus. Therefore, we cannot exclude that we did not measure only uptake in the pituitary, but also in the basal hypothalamus. In other ongoing studies, by also acquiring magnetic resonance (MR) images for better anatomical referencing, we aim to evaluate this relevant topic.

### 4.3. No Significant Uptake of ^68^Ga-NODAGA-Exendin-4 in the Brain

Apart from uptake in the pituitary, we observed that there is no significant uptake of ^68^Ga-NODAGA-exendin-4 in other parts of the brain of subjects with obesity. Although expression of GLP-1 receptors is well documented in cortical and subcortical brain areas [4,8,9,10], the lack of uptake may be explained by the low lipophilicity and/or large molecular weight of the PET tracer. Indeed, other gallium-68-labelled tracers such as ^68^Ga-dotatate (which binds preferentially to the somatostatin receptor-2) also showed no uptake in the brain (being much smaller than exendin and containing less charges), while these somatostatin receptors are expressed throughout the brain in deep layers of cortical cortex as well as in the cerebellum [42]. If labeling with the radionuclide gallium-68 indeed hinders brain uptake, development of e.g., fluorine-18 labelled PET tracers derived from exendin-4 may allow the visualization of GLP-1 receptors in the brain. Interestingly, recent animal studies using ^18^F-AlF-NOTA-MAL-Cys^39^-exendin-4 showed indeed some in-vivo binding to GLP-1 receptors in the brain of rats [43,44]. However, the specific binding in brain was very low, which precluded use of this tracer in brain studies in humans. Finally, a recent study evaluated another fluorine-18 labelled PET tracer derived from exendin-4 (^18^F-FB(ePEG12)12-Exendin-4) in humans; this tracer also did not show any brain uptake [45]. So, it is questionable if this approach will be successful in future human PET studies aiming to visualize GLP-1 receptors in the brain (see also below).

### 4.4. Uptake of GLP-1 Receptor Agonists in the Brain

As already mentioned, administration of GLP-1 analogues induces central effects, which suggests that GLP-1 analogues may pass the BBB. Indeed, several studies suggested that GLP-1 analogues may pass the BBB [46,47]. Also, in a placebo-controlled clinical study in PD, in which half of the PD patients were treated for almost 1 year with the GLP-1 analogue exenatide [27], exenatide was detectable in the cerebrospinal fluid (CSF). Conversely, a clinical study in type 2 diabetes showed that the transfer from blood to CSF of the GLP-1 analogue liraglutide was only minimal [48].

Successfully developed radiotracers for brain studies in humans are transported actively over the BBB, or diffuse rapidly into the brain [49]. For example, the PET tracer ^18^F-DOPA is taken up fast by the large neutral amino acid transport carrier-mediated system of the BBB [50]. Alternatively, successful brain PET/SPECT tracers should not be transported rapidly from the brain back to the blood, e.g., by efflux via the P-glycoprotein (PgP) expressed in the BBB [51].

However, whether, and what extent, GLP-1 analogues penetrate the BBB is an important point of discussion in research on the mechanism of action of this class of drugs [9,23]. For example, the GLP-1 analogue liraglutide (which is marketed both for treatment of type-2 diabetes and obesity) was injected intraperitoneally (i.p.) in mice, and later on the levels of this drug were measured in the brain. Interestingly, there were no significant levels of liraglutide found 5 min post-injection in the brains of mice administered with a 2.5, 25 or 250 nmol/kg dose of the peptide. Also, at higher doses (25–250 mg/kg bodyweight), no significant levels of liraglutide were found 5 min post i.p., but only at later time-points [46]. This may indicate that the passage of GLP-1 analogues (and possibly related radiotracers) via the BBB is not rapid, and probable not by active transport. Moreover, to evaluate the mechanism by which liraglutide is able to reduce body weight, mice were injected subcutaneously with liraglutide labeled with a fluorescent probe [52]. Interestingly, liraglutide labelling was highly abundant within parts of the hypothalamus, including the arcuate nucleus and paraventricular nucleus, as well as the median eminence, but not in other brain areas. No liraglutide signal was observed in the nucleus tractus solitarius, which could indicate that peripheral administration of liraglutide does not directly engage the GLP-1 receptor in this brain region.

Interestingly, recent evidence indicates that peripheral administrated peptides (e.g., GLP-1) may enter the brain via transcytosis across so-called tanycytes (which are classified as astroglia). These tanycytes are located around the floor of the third ventricle (in the area of the median eminence and basal hypothalamus), and by transport via these tanycyte peptides can enter the third ventricle (CSF), but also distant brain structures lining the ventricles (for a recent review see Garcia-Caceres et al. [33]). Importantly, a recent study by Gabery and co-workers demonstrated that tanycytes express GLP-1 receptors. They also showed that the GLP-1 analogue semaglutide, when administrated peripherally, do not enter the brain by general BBB permeability, but via the tanycytes [9]. Regarding neuro- PET imaging, if PET tracers derived from exendin-4 may enter the brain also via transcytosis by tanycytes, this approach may not lead to a successful development of neuro PET tracers.

### 4.5. Final Remarks and Limitations

In sum, we only observed uptake in the brain of ^68^Ga-NODAGA-exendin-4 in the pituitary area of subjects with obesity and it is becoming more likely that (part) of the mechanism of actions of GLP-1 receptor agonists and PET tracers on brain functions might be due to (slow) uptake by tanycytes, located in the median eminence/basal hypothalamus. To shed more light on this topic, it may be relevant to determine whether ^68^Ga-NODAGA-exendin-4 binds in-vivo in humans only in the pituitary, or also in the median eminence/basal hypothalamus. Additionally, it still cannot be excluded that the central effects of GLP-1 analogues are (partly) induced via peripheral mechanisms and/or by direct uptake via the pituitary/basal hypothalamus.

If future novel PET tracers derived from exendin-4 do show rapid and intense uptake in the brain, it is unlikely that this brain uptake is only by the tanycytes. If this happens, it is more likely that they pass the BBB by an active mechanism or diffuse rapidly through the BBB. Furthermore, it may be relevant to determine whether tracers derived from exendin-4, like ^68^Ga-NODAGA-exendin-4, are a substrate for the efflux transporter PgP.

We only studied ^68^Ga-NODAGA-exendin-4 in obese subjects. We therefore cannot exclude that it is possible to visualize GLP-1 receptors in the brain in disorders in which the BBB function is severely disrupted. Finally, we only studied a small number of subjects. Therefore, our findings are in need for replication.

## 5. Conclusions

In conclusion, we have shown that in subjects with obesity there is no significant uptake of ^68^Ga-NODAGA-exendin-4 in the parts of the brain located within the BBB. Therefore, ^68^Ga-NODAGA-exendin-4 PET cannot be used to analyze GLP-1 receptors in the brain of obese subjects. However, there is clear uptake of the tracer in the pituitary, which offers the unique opportunity to evaluate the role of GLP-1 receptor expression in the pituitary in health and disease.

## Figures and Tables

**Table 1:** Demographic and anthropometric characteristics of the 10 included subjects.

Sex, Woman (%)	7 (70%)
Age (years)	53 ± 5.5
Weight (kg)	113 ± 13
BMI (kg/m^2^)	39 ± 4.4

Data are expressed as number of cases (%) or mean ± SD.

**Table 2:** Uptake values of ^68^Ga-NODAGA-exendin-4 (*n* = 10).

	SUV_mean_	SUV_max_
Pituitary	1.7 ± 0.6 (0.9–2.7)	4.3 ± 2.3 (1.4–9.1)
Whole brain	0.01 ± 0.01 (0.00–0.02)	0.17 ± 0.10 (0.01–0.32)
Pancreas	5.5 ± 1.8 (2.3–8.1)	10.3 ± 3.0 (5.0–15.5)
Blood pool	1.5 ± 0.2 (1.2–2.0)	3.3 ± 0.6 (2.4–4.4)
Liver	0.67 ± 0.17 (0.26–0.96)	2.1 ± 0.6 (1.3–3.3)
SAT	0.20 ± 0.05 (0.14–0.29)	0.65 ± 0.16 (0.38–0.91)

Data are mean values ± standard deviation (range). SAT: subcutaneous adipose tissue, SUV: standardized uptake value.

## Data Availability

The data presented in this study are available on request from the corresponding author.
